# Thrombophilia and Pregnancy Complications

**DOI:** 10.3390/ijms161226104

**Published:** 2015-11-30

**Authors:** Louise E. Simcox, Laura Ormesher, Clare Tower, Ian A. Greer

**Affiliations:** 1 Maternal and Fetal Health Research Centre, Institute of Human Development, Faculty of Medical and Human Sciences, University of Manchester, Hathersage Road, Manchester M13 9WL, UK; Clare.Tower@cmft.nhs.uk; 2 St. Mary’s Hospital, Central Manchester University Hospitals NHS Foundation Trust, Manchester Academic Health Science Centre, Manchester M13 9WL, UK; Laura.Moncreiffe@cmft.nhs.uk; 3 The University of Manchester, Oxford Road, Manchester M13 9PL, UK; ian.greer@manchester.ac.uk

**Keywords:** thrombophilia, pregnancy complications, heparin, fetal growth restriction, recurrent pregnancy loss

## Abstract

There is a paucity of strong evidence associated with adverse pregnancy outcomes and thrombophilia in pregnancy. These problems include both early (recurrent miscarriage) and late placental vascular-mediated problems (fetal loss, pre-eclampsia, placental abruption and intra-uterine growth restriction). Due to poor quality case-control and cohort study designs, there is often an increase in the relative risk of these complications associated with thrombophilia, particularly recurrent early pregnancy loss, late fetal loss and pre-eclampsia, but the absolute risk remains very small. It appears that low-molecular weight heparin has other benefits on the placental vascular system besides its anticoagulant properties. Its use is in the context of antiphospholipid syndrome and recurrent pregnancy loss and also in women with implantation failure to improve live birth rates. There is currently no role for low-molecular weight heparin to prevent late placental-mediated complications in patients with inherited thrombophilia and this may be due to small patient numbers in the studies involved in summarising the evidence. There is potential for low-molecular weight heparin to improve pregnancy outcomes in women with prior severe vascular complications of pregnancy such as early-onset intra-uterine growth restriction and pre-eclampsia but further high quality randomised controlled trials are required to answer this question.

## 1. Introduction

Pregnancy alters the haemostatic system into a hypercoagulable state, which increases throughout pregnancy and is maximal around term. Most notably there is a significant change to coagulation, with increased factor VII, VIII, X and von Willebrand factor activity and marked increases in fibrinogen. [1]. Thrombin generation markers such as prothrombin F1 and 2 and thrombin-antithrombin (TAT) complexes are also increased [2]. There is also a marked decrease in anticoagulant activity including reduced protein S levels and acquired activated protein C resistance [1]. Fibrinolytic activity is also reduced with plasminogen activator inhibitor type 1 (PAI-1) levels increased by five-fold and increases in placentally-derived plasminogen activator inhibitor type 2 (PAI-2), particularly during the third trimester [3]. These changes in the haemostatic system act as a physiological “safety net” for the peripartum period, but can predispose both the mother and fetus to complications during the pregnancy. For the mother, this risk begins from the point of conception well into the postnatal period, with recent data suggesting that the risk extends to at least 12 weeks [4]. For the fetus during pregnancy, risks include pre-eclampsia, placental abruption, fetal growth restriction (FGR), late and recurrent early miscarriage, intrauterine death and stillbirth. The risks are inherently higher in women with acquired or inherited thrombophilia, but at present routine screening for these disorders is not routinely recommended in the absence of venous thromboembolism. Further, the value of screening those with pregnancy complications is uncertain because of limitations in knowledge on their contribution to such pregnancy complications and lack of evidence for effective intervention. In addition, some of the tests themselves are imprecise, for example blood levels of factors including antithrombin, protein C and protein S can be affected by circulating oestrogen levels, pregnancy and the postpartum state as well as intercurrent illness, in addition to the clinical implications of both positive and negative results being often misunderstood. Even with functional assays, no single method of testing will detect all defects, and if a true association is found, the absolute risk of an adverse outcome is low, based on different mechanistic pathologies. Due to the growing pregnant population and successful artificial reproductive technologies, many women are now older and have more medical complications when embarking on pregnancy, hence testing for heritable thrombophilias in women with previous pregnancy complications is becoming increasingly common as a practice despite the limitations described above.

Low dose aspirin and low molecular weight heparin (LMWH) have proven their effectiveness in increasing live birth rates in the setting of antiphospholipid syndrome. However, their use in the context of inherited thrombophilia and pregnancy complications is less well established. There is however increasing evidence for the use of heparin in women with pregnancy complications mediated by the placenta, selected by previous pregnancy outcome and not by thrombophilic defect [5]. Due to their excellent safety record, these treatments have been offered to women at high risk of adverse pregnancy outcome in advance of the scientific evidence becoming available for their use. Clinicians proposed these treatments based on biological plausibility and extrapolation from antiphospholipid syndrome, and in the context of aspirin, its use in the prevention of pre-eclampsia and small for gestational age infants [6]. Biological studies have now demonstrated that heparin has other beneficial effects on the placenta in addition to the anticoagulant properties mediated through its interaction with antithrombin. These include increased tissue factor pathway inhibitor, angiogenesis on the antiphospholipid antibody-inhibited human endometrial endothelial cell, and reduced trophoblast apoptosis, complement activation and platelet aggregation [6].

## 2. Inherited Thrombophilias

Several studies have suggested an association between adverse pregnancy outcome and heritable thrombophilias, and homozygosity for the thermolabile mutation of methylene tetrahydrofolate reductase (MTHFR) causing homocysteinaemia. It is hypothesised that thrombophilia may cause placental insufficiency due to placental vascular thrombosis. Other studies do not support this association and overall the evidence is weak. The arguments against the current hypothesis of placental vascular thrombosis conclude that if hypercoagulability was the causal association, then why is pregnancy loss and stillbirth not higher in women with a previous history of venous thromboembolism than in controls. Evidence from meta-analysis of case-control studies where women with pregnancy complications were compared to those without concludes that there is an increased prevalence of thrombophilia in those women with complications, but the magnitude of the association is modest suggesting that this is a contributory factor rather than a primary cause [7,8,9]. A comprehensive systematic review on the association is summarised in Table 1 [9]. Although late pregnancy loss appeared to be most strongly associated with protein S deficiency, numbers were small (*n* = 15), and reliable diagnosis of this condition is often inaccurate. The odds ratios reported were highest for factor V Leiden and FII 20210A (Prothrombin gene) heterozygotes and in particular related to later pregnancy complications, such as second trimester loss, pre-eclampsia, fetal growth restriction and placental abruption rather than recurrent first trimester loss. As most reports were retrospective it is important to consider prospective studies. Rodger *et al.* [7] performed a meta-analysis of prospective cohort studies to estimate the association of maternal factor V Leiden or prothrombin gene carrier status and placenta-mediated pregnancy complications. Overall, the odds ratio for pregnancy loss in women with factor V Leiden was 1.52 (95% CI 1.06–2.19), which translates into a low absolute rate for pregnancy loss (4.2%), compared to a 3.2% pregnancy loss rate in women without factor V Leiden. There was no significant association between factor V Leiden and prothrombin gene and the composite outcomes of pre-eclampsia, small for gestational age (SGA), pregnancy loss or placental abruption with pooled odds ratios of 1.08 (95% CI 0.87–1.52) and 1.27 (95% CI 0.94–1.71), respectively. Due to the relatively low prevalence of prothrombin gene mutation, the study was not adequately powered to identify important increases in the risk of pregnancy loss in those women. In particular, the study only had adequate power to detect an absolute increase in risk of >4% from the observed control group event rate of 3.6%, *i.e.*, it could not exclude a doubling of risk from 3.6% to 7.2%. Following a recent prospective cohort study by Rodger *et al*. [10] in which 7343 women were recruited to examine whether or not factor V Leiden or prothrombin gene mutations were associated with placenta-mediated complications (pre-eclampsia, small for gestational age fetus <10th centile, pregnancy loss or placental abruption), this meta-analysis was updated. Findings from the cohort study revealed that of the 507 women (6.9%) with factor V Leiden and/or prothrombin gene mutation, 11.64% suffered a placenta mediated complication compared to 11.23% in the cohort without detectable thrombophilia giving a non-significant absolute risk difference of 0.41% and an odds ratio close to one. It is important to note however that the study was only powered to detect a difference in odds ratio of >1.5 and not an increase in absolute risk. The study also had to analyse composite pregnancy outcomes as one group as it was underpowered for the individual analyses of placental abruption, pregnancy loss and pre-eclampsia. By combining the results of this study with the previous meta- analysis, the authors finally concluded a very weak association between factor V Leiden and pregnancy loss, which translates to a very small absolute increased risk from 2.79% to 3.58% in factor V Leiden carriers [10]. Limitations include that the current meta-analysis could not address whether inherited thrombophilia was associated with early pregnancy loss as most women were recruited in the late first or second trimester. In the cohort study, 77% of the recruited women were Caucasian and so the results may not be applicable to a different mixed ethnicity population. Some studies have found an association between MTHFR homozygosity and recurrent pregnancy loss, and others have not. The above meta-analysis has suggested that there is no association and this may be because folic acid replacement suppresses the phenotypic expression of this polymorphism. To summarise these findings, the predictive value of a positive thrombophilia screening result for recurrence of later pregnancy complications cannot be determined with accuracy.

**Table 1:** Association between Thrombophilia and Pregnancy Complications.

Thrombophilia	Frequency	Early (Recurrent) Pregnancy Loss	Late Loss	Pre-Eclampsia	Placental Abruption	Intrauterine Growth Restriction
Factor V Leiden (Homozygous)	0.06	2.71 (1.32–5.58)	1.98 (0.40–9.69)	1.87 (0.44–7.88)	8.43 (0.41–171.20)	4.64 (0.19–115.68)
Factor V Leiden (Heterozygous)	4	1.68 (1.09–2.58)	2.06 (1.10–3.86)	2.19 (1.46–3.27)	4.70 (1.13–19.59)	2.68 (0.59–12.13)
Prothrombin Gene Variant (Heterozygous)	2	2.49 (1.24–5.00)	2.66 (1.28–5.53)	2.54 (1.52–4.23)	7.71 (3.01–19.76)	2.92 (0.62–13.70)
MTHFR C677T (Homozygous)	5–25	1.40 (0.77–2.55)	1.31 (0.89–1.91)	1.37 (1.07–1.76)	1.47 (0.40–5.35)	1.24 (0.84–1.82)
Antithrombin Deficiency	0.07	0.88 (0.17–4.48)	7.63 (0.30–196.36)	3.89 (0.16–97.19)	1.08 (0.06–18.12)	NA
Protein C Deficiency	0.3	2.29 (0.20–26.43)	3.05 (0.24–38.51)	5.15 (0.26–102.22)	5.93 (0.23–151.58)	NA
Protein S Deficiency	0.2	3.55 (0.35–35.72)	20.09 (3.70–109.15)	2.83 (0.76–10.57)	2.11 (0.47–9.34)	NA
Lupus Anticoagulant	2 *	NA	2.4 (0.8–7.0)	1.5 (0.5–4.6)	NA	NA
Anticardiolipin Antibodies		3.4 (1.3–8.7)	3.3 (1.6–6.7)	2.7 (1.7–4.5)	1.42 (0.42–4.77)	6.9 (2.7–17.7)

* incidence for Antiphospholipid Antibodies (Lupus Anticoagulant, Anticardiolipin Antibodies); From Robertson *et al.* [9]; NA—Not Available.

## 3. Acquired Thrombophilia

The most commonly reported form of acquired thrombophilia is the antiphospholipid syndrome (APS). This is usually diagnosed in a patient retrospectively following an arterial or venous thrombosis (or a pregnancy complication in women). The diagnostic criteria for this condition are strict and patients must be found to have antiphospholipid antibodies (anticardiolipin antibodies (aCL), and/or lupus anticoagulant (LA); and/or anti-β-2-glycoprotein I antibodies (aβ2-GPI)), persisting for two or more separate occasions, at least 12 weeks apart. This is because aPL (antiphospholipid antibodies) are found in 3%–5% of those without thrombosis or obstetric morbidity and the antibodies themselves are often transient, associated with infection or drugs [11]. Of women with recurrent pregnancy loss (defined as three or more consecutive early pregnancy losses), 10%–20% have detectable aPL [12]. Late pregnancy complications relate to vascular complications in the placenta caused by aPL resulting in placental insufficiency and include pre-eclampsia, fetal growth restriction, pre-term delivery and stillbirth. A meta-analysis suggested that the antibodies most strongly associated with late second trimester pregnancy loss and venous thromboembolism were those directed against domain I of aβ2-GPI to cause inhibition of trophoblast invasiveness [13]. aCL antibodies were found to be associated with both early and late recurrent pregnancy loss and pre-eclampsia. Subsequently, a more recent meta-analysis in 2011 concluded that antiphospholipid antibodies appear to be most consistently associated with late fetal loss, with LA being the most strongly and consistent antibody associated with placenta mediated pregnancy complications. They also concluded that there was insufficient data in the literature to prove a significant association between aβ2-GPI and pregnancy morbidity [14]. In addition, Annexin A5 (placental anticoagulant protein) is abundant on the membranes of placental syncytiotrophoblasts and is decreased in the presence of aPL. Annexin is an anti-coagulant and displacement of this protein from trophoblasts mimics a procoagulant state through acceleration of coagulation reactions [15]. A combination of polymorphisms in the core promoter of annexin A5 gene (ANXA 5, located at 4q27) termed as M2 haplotype has found to be associated with increased risk of recurrent early pregnancy loss and pregnancy-related hypertensive disorders [16,17,18].

## 4. Biological Plausibility for Low Molecular Weight Heparin Use

Some obstetricians now offer a thromboprophylactic dose of LMWH to women with a previous history of pregnancy complications, particularly recurrent miscarriage or previous stillbirth. This is either based upon laboratory evidence of a thrombophilic defect or findings of thrombotic changes from placental histology specimens in the affected pregnancy. Risks of treatment with LMWH are low, but the evidence base to guide decision making upon starting this treatment, particularly late pregnancy complications, is lacking from adequately powered randomised controlled trials (RCTs). The strongest evidence for the use of aspirin and LMWH together is in the presence of antiphospholipid syndrome and recurrent pregnancy loss; as this combination has been shown improve live birth rates. However, to obtain the maximum benefit from these therapeutic strategies, medication must be started early on in pregnancy (ideally six weeks gestation), before primary trophoblast invasion is complete, although there is still benefit to be gained up until 18 weeks gestation whereby the second phase of invasion into the myometrium is completed [19].

Most of the evidence we have for treating women with LMWH for heritable thrombophilia and pregnancy complications is based on the plausibility of it being effective in biological studies looking at recurrent pregnancy loss in APS and implantation failure in IVF (*in vitro* fertilisation). Most studies suggest that the mechanism by which LMWH exerts its beneficial effects is more complicated than by simple thrombin inhibition alone. This is highlighted in a study by An *et al.* [20] using a murine high-risk pregnancy model that demonstrated a significantly reduced abortion rate in mice treated with LMWH, an indirect factor Xa inhibitor, which exerts its action through antithrombin. This finding was not replicated when the mice were treated with lepirudin (direct thrombin inhibitor) or fondaparinux (indirect specific Xa inhibitor). Another study has demonstrated that LMWH also blocks activation of complement initiated by aPL-IgG targeted to decidual tissues both *in vivo* and *in vitro* [21]. Other mechanisms by which heparin may act to improve implantation and subsequent pregnancy outcome include an anticoagulant effect and increase in tissue factor pathway inhibitor, direct effects on the trophoblast such as reduced apoptosis and enhanced endometrial angiogenesis, and modulation of the immune system [22].

## 5. *In Vitro* Fertilisation (IVF) and Implantation Failure

During implantation, progesterone induces oestradiol-primed human endometrial stromal cells to undergo decidualisation, which protects against bleeding due to endometrial capillaries being invaded by implanting cytotrophoblasts [23].There is a recruitment of factors to promote haemostasis including upregulated expression of tissue factor, the primary initiator of haemostasis via thrombin generation, and plasminogen activator inhibitor type 1 (PAI1, SERPINE 1), which inactivates tissue-type plasminogen activator (t-PA, PLAT), the predominant agent in fibrinolysis. The role of thrombophilia in recurrent implantation failure following IVF treatments is thought to be through mechanisms similar to those seen in recurrent miscarriage and has been the focus of research efforts. It has been hypothesised that invasion of maternal vessels by syncytiotrophoblast can be affected by localised thrombosis at the implantation site, leading to IVF failure [22]. In addition, the thrombomodulin-protein C system is essential (as an inhibitor of coagulation and fibrinolysis), to prevent over production of tissue factor which in turn leads to generation of thrombin and ultimately fibrin degradation products that are toxic to trophoblast cells [24]. The potential for heparin to positively affect implantation emphasises the important role of the haemostatic system in this process [6]. Qublan *et al.* [25], randomised 83 women with a history of three or more previous IVF failures and at least one thrombophilic defect to either treatment with enoxaparin 40 mg daily or to placebo. Both treatments were commenced on the day of embryo transfer and continued until delivery or until fetal demise was diagnosed. Overall the live birth rate was 23.8% in the heparin treated group *vs.* 2.8% in the placebo group (*p* ≤ 0.05). These findings are similar to previous uncontrolled trials of antithrombotic therapy with unfractionated heparin (UFH) in women with repeated IVF-embryo transfer (ET) failures and thrombophilia [25]. In another study examining inherited thrombophilic defects, women with at least two failed IVF or intra-cytoplasmic sperm injection (ICSI) treatments were screened for factor V Leiden, prothrombin gene mutation or MTHFR C677T and excluded if antiphospholipid antibodies were detected [26]. Participants received LMWH from controlled hyperstimulation until the β-hcg test. The only significant finding was that the pregnancy rate in women >36 years was higher in those receiving LMWH when compared to those not receiving LMWH. There was no significant difference found between the presence/absence of thrombophilia, but only 32% of the study population had a thrombophilia marker, largely MTHFR C677T homozygotes (25%). In these studies it is clear that LMWH prophylaxis appears to reduce the risk of implantation failure, increases live birth rates and reduces abortion rates, but the numbers in studies are too small to extrapolate and give guidance to clinicians in prescribing for specific inherited or acquired thrombophilic defects.

## 6. Early Pregnancy Complications

This area of research in mainly focused on antiphospholipid syndrome and recurrent miscarriage for which there is an established “thrombotic” link. Only 1%–2% of couples will be diagnosed with recurrent miscarriage, this is in contrast to sporadic miscarriage related to fetal chromosome abnormalities which is relatively more common, (approximately 25% of couples experience a single event) [27]. Recurrent miscarriage causes considerable psychological morbidity both for the woman and her partner. It is hypothesized that the causes of recurrent miscarriage can be related to an exaggerated haemostatic response in pregnancy [28], and this is evident in cases of antiphospholipid syndrome-related pregnancy loss. In antiphospholipid syndrome, there is evidence of placental infarction in histology specimens [29], increased thrombin generation and improved live birth rates in affected cases treated with antithrombotic therapy [30]. Given this strong causal association, pregnancy outcome in untreated women with aPL and a history of recurrent miscarriage is poor. Two RCTs have reported that a combination of low-dose aspirin and UFH improves live birth rates in women with antiphospholipid syndrome in comparison to those women treated with aspirin alone [31,32]. Some studies have tried to challenge these findings by suggesting that low-dose aspirin alone is just as effective, however there are considerable flaws in their methodologies. In one of these studies by Farquharson *et al.* [33], women were included with low positive titres for aCL antibodies, assigned to treatment at a late stage in the first trimester and there was a 25% crossover between treatment groups. The evidence to support a decision of choosing UFH rather than LMWH or vice versa for prevention of recurrent pregnancy loss is not established, but the safety profile of LMWH makes it a more attractive choice. It is also important to remember that in these women with aPL, a small proportion of them will remain at risk for complications in the late second and third trimesters of pregnancy due to underlying placental vasculopathy. In one prospective series of 150 treated women with antiphospholipid syndrome, there was an increased risk of hypertension (17%), fetal growth restriction (15%), antepartum haemorrhage (7%), and pre-term delivery (24%) [34].

Several studies are now examining the causal association between the Annexin M2 haplotype and pregnancy complications such as recurrent pregnancy loss and pre-eclampsia. Reduced annexin A5 expression levels on placental syncytiotrophoblasts have been described both in women with aPL and also in pre-eclamptic patients immunohistochemically whereby it contributes to localised thrombosis at the feto-maternal interface and subsequent fetal growth restriction [18]. Frequency of the M2 haplotype has been found to be significantly higher in patients with recurrent pregnancy loss in several different ethnic populations, than in healthy controls. Bogdanova *et al.* [17] demonstrated that the M2 haplotype of the annexin gene reduced the *in vitro* activity of the ANXA5 promoter to 37%–42% of the normal level. This was associated with a >2-fold higher recurrent pregnancy loss risk in 70 recurrent pregnancy loss patients when compared to a group of “unselected” pregnancy controls (OR, 2.42; 95% CI, 1.27–4.58). In a similar study by Tiscia *et al*. [16], they found the M2 haplotype to be associated with a three-fold higher recurrent pregnancy loss risk and a two-fold increase in pregnancy related hypertensive disorders, but it was not found to be associated with an increased risk of intra-uterine fetal death. However, the numbers of cases in the intra-uterine fetal death group were half of those in the other two groups and so this may have influenced the findings. The largest cohort study so far, involving 500 white European couples demonstrated a significantly increased M2 allele frequency in patients with early recurrent pregnancy loss but not in those who had experienced late second trimester fetal loss (OR 1.47, 95% CI 1.03–2.09) [35]. The risk appeared to increase with increasing number of M2 alleles and in the presence of other risk factors such as factor V Leiden and prothrombin gene mutations, but the study was not adequately powered to prove these associations. One retrospective case-control study has challenged these findings and found the M2 haplotype not to be associated with recurrent pregnancy loss [15]. Explanations for these differences could be due to differing selection of appropriate fertile healthy controls and also whether maternal or paternal carriership of the haplotype influences outcomes. Most studies suggest an equal contribution of paternal and maternal alleles in the risk of recurrent pregnancy loss [18,35] therefore implicating a placental rather than a maternal genotype. However, a study with a murine ANXA5 knockout model has shown the role of only maternal ANXA5 production in pregnancy maintenance [36]. It is clear that additional studies are required to investigate the contribution of the M2 haplotype in pregnancy related fetal and early pregnancy loss.

## 7. Late Pregnancy Complications

Late pregnancy complications are more commonly associated with acquired thrombophilia and systemic lupus erythematosus rather than inherited thrombophilia. The mechanism of fetal loss in antiphospholipid syndrome is due to attachment of aPL to trophoblast cells, resulting in abnormal placentation. Most studies have also found an association with positivity for aCL and early-onset pre-eclampsia. Women with antiphospholipid syndrome are also at risk (increased by approximately 30%) of fetal growth restriction and often require regular growth scans throughout pregnancy. A recent meta-analysis in 2011 examined the association between antiphospholipid antibodies and placenta mediated complications (pre-eclampsia, intra-uterine growth restriction, late fetal loss and placental abruption) [14]. There were 28 studies in the final analysis but unfortunately most of these were small case-control studies and so results were given as odds ratios and the question of whether or not there is an actual increase in the risk of these complications with antiphospholipid antibodies remains unanswered. They found all three antibodies (LA, aCL and aβ2-GPI) to be associated with pre-eclampsia and late fetal loss, and LA was the most strongly associated with these complications (OR of 2.34, 95% CI 1.81–4.64 and 4.73, 95% CI 1.08–20.81 respectively for pre-eclampsia and late fetal loss). There are certain factors that make it difficult to interpret studies looking at the association between antiphospholipid antibodies and late pregnancy complications. These include heterogeneity in study designs, inclusion of women with low positive aPL levels in those women considered positive, different definitions of late pregnancy loss amongst studies, and population heterogeneity with respect to the clinical phenotype of antiphospholipid syndrome. It is also difficult to prove a definite association between inherited thrombophilia and placenta mediated complications due to different classifications of fetal growth restriction based on either birthweight percentile or absolute birthweight and different severities of pre-eclampsia being classified as one outcome group. As a result of this, studies are comparing different fetal populations when assessing neonatal outcomes. In the large Danish National Birth Cohort study, factor V Leiden, prothrombin G20210A and MTHFR C677T mutations were assessed for the risk of severe pre-eclampsia, fetal growth restriction, early preterm delivery and a composite of these [8]. This involved a nested case-control study of 2032 cases and 1851 random controls. Factor V Leiden increased the risk of composite outcome (OR 1.4, 95% CI 1.1–1.8), severe preeclampsia (OR 1.6, 95% CI 1.1–2.4), fetal growth restriction (OR 1.4, 95% CI 1.1–1.8) and abruption (OR 1.7, 95% CI 1.2–2.4). Prothrombin G20210A was not associated with any adverse outcome and MTHFR C677T was associated with severe preeclampsia (OR 1.3, 95% CI 1.1–1.6). A meta-analysis has found that factor V Leiden is associated with a twofold increased risk of late unexplained fetal loss and a fourfold increased risk of late second trimester loss [9]. However, due to limitations on the available data within the systematic review, pregnancy outcomes for both homozygous and heterozygous factor V Leiden carriers were analysed as one group. A recent prospective cohort study and subsequent updated meta-analysis has now demonstrated that carriership for either factor V Leiden or prothrombin gene mutation is not associated with adverse pregnancy outcomes apart from a weak association between factor V Leiden and pregnancy loss [10]. Other inherited thrombophilias associated with stillbirth include antithrombin deficiency (OR 5.2, 95% CI 1.5–18.1), protein C deficiency (OR 2.3, 95% CI 0.6–8.3), and protein S deficiency (OR 3.3, 95% CI 1.0–11.3) [37].

## 8. Management

There is evidence in the case of antiphospholipid syndrome, for a beneficial effect of aspirin and heparin to prevent recurrent miscarriage and fetal loss. There is however no benefit of LMWH treatment in preventing early recurrent pregnancy loss in women without thrombophilia. Kaandorp *et al.* [38] randomised 364 women with unexplained recurrent miscarriage into three groups: low-dose aspirin and LMWH, aspirin alone or placebo. The main outcome measure was live birth rate which did not differ significantly between the three groups. In addition, the live birth rate in the placebo group of 67% was higher than that in the aspirin only group (61.6%), giving a non-significant absolute risk difference of 5.4% (95% CI −18.6–7.8). Visser *et al.* [39] conducted a randomised double-blind trial on 207 women with three or more first trimester miscarriages, two or more second trimester miscarriages or one third trimester fetal loss combined with one first trimester miscarriage. The live birth rate was 71% (RR 1.17, 95% CI 0.92–1.48) for LMWH and placebo and 65% (RR 1.08, 95% CI 0.83–1.39) for LMWH and aspirin compared with aspirin alone (61%). However, the trial was discontinued in advance of its completion due to slow recruitment. The study did also not have enough power to exclude a benefit of enoxaparin treatment, *i.e.*, it did not have enough power to exclude an increase in the live birth rate of 10% (61%–71%) with enoxaparin. Lastly, Schleussner *et al.* [40] randomised 449 women with two consecutive early miscarriages or one late miscarriage to placebo and heparin treatment *vs.* placebo. The main outcome measure of the study was ongoing pregnancy at 24 weeks gestation, which did not differ significantly between the two groups (absolute risk difference of 1.1%). Live birth rates were 86% in the intervention group *vs.* 86.7% in the placebo arm giving a non-significant risk difference of 0.7% (95% CI −7.3–5.9). In heritable thrombophilia, there is no proven therapeutic intervention, except for the role of low-dose aspirin in the prevention of pre-eclampsia. Other trials have looked at the potential role of heparin in women with a history of previous pre-eclampsia and thrombophilia, and found that it reduced the risk of early-onset pre-eclampsia <34 weeks. The FRUIT RCT found that adding LMWH to aspirin before 12 weeks gestation reduced the risk of recurrent early onset pre-eclampsia in women with inherited thrombophilia and prior delivery for pre-eclampsia/fetal growth restriction before 34 weeks [41]. However other than reduced steroid use (for preterm delivery) in the control group there was no difference in clinical outcome. In the pilot randomised NOH-PE trial [42], women who had experienced severe pre-eclampsia in their first pregnancy were randomised to enoxaparin *vs.* no enoxaparin. Of these patients, 12%–16% also had another thrombophilia but these frequencies did not differ between intervention and non-intervention groups. Composite end-points (pre-eclampsia, placental abruption, birth weight <5th centile and fetal loss after 20/40) were found to be reduced in the enoxaparin group but the only one that reached significance was recurrence of pre-eclampsia. In the TIPSS trial [43], women were recruited with a history of thrombophilia and increased risk of placental-mediated pregnancy complications and/or venous thromboembolism. They were subsequently randomised to LMWH (dalteparin) *vs.* no LMWH. The trial concluded no significant difference in pregnancy loss or placental-mediated complications with LMWH. However, most of the women recruited were factor V Leiden heterozygotes (60%) with <10% aPL, 30% Prothrombin, and approximately 15%–20% of protein C, protein S and antithrombin deficiencies combined. Although the two groups were comparable in the frequencies of thrombophilic defects, it is likely the only group that reliable conclusions can be drawn from is factor V Leiden heterozygotes, given low representation of other thrombophilic defects. Although a combination of LMWH with aspirin appears to have improved perinatal outcome in some women with a poor obstetric history and inherited thrombophilia, there is lack of clear evidence from RCTs. New evidence is emerging to suggest the benefit of treatment with LMWH to prevent placenta mediated complications in women selected by previous pregnancy outcome alone. Rodger *et al.* [5] performed a recent meta-analysis of RCTs looking at LMWH *vs.* no LMWH for the prevention of recurrent placenta-mediated complications. Primary outcome was a composite measure of pre-eclampsia, birth of a growth restricted infant <10th centile, pregnancy loss >20 weeks or placental abruption. Overall 18.7% of women being given prophylactic LMWH had recurrent placenta mediated complications *vs.* 42.9% of women with no LMWH (*p* < 0.01). These results have to be interpreted carefully as participants with thrombophilic defects were not excluded from the studies and there was a co-intervention of low-dose aspirin in 50% of participants. There were also larger relative risks for more severe pregnancy complications such as severe early onset pre-eclampsia and fetal growth restriction <5th percentile. It may be that LMWH would be of maximal benefit in a subgroup of patients with prior severe pregnancy complications.

## 9. Conclusions

Currently, routine screening for thrombophilic defects is not recommended in women without previous pregnancy complications. However, prevention of placenta-mediated complications such as recurrent miscarriage, early and late-onset fetal growth restriction and stillbirth remains a major and topical health issue. It is currently not clear whether the disease process itself and the natural history of the disease is completely understood if we look at inherited thrombophilia relating to late pregnancy complications and the role of the M2 Haplotype in recurrent pregnancy loss. Although we know that there are safe and effective tests for thrombophilic defects, these are often difficult to perform accurately and misinterpreted, especially if undertaken in the pregnant or early postpartum period. Further, while screening might identify women with thrombophilia, there is no proven intervention that would follow such screening.

Women with both inherited and acquired thrombophilia are at increased risk of developing both early and late complications in pregnancy. However, the absolute risk of these adverse outcomes remains low. With respect to inherited thrombophilias, the available studies are largely underpowered to detect significant outcomes due to the low prevalence of these defects in the general population. In studies looking at late pregnancy complications, classification and definitions of fetal growth restriction and “severe pre-eclampsia” are non-specific and not clearly stated and hence the comparisons are made between heterogeneous populations. Turning to treatment, aside from the prevention of recurrent pregnancy loss and venous thromboembolism in antiphospholipid syndrome, there is insufficient evidence to guide the use of heparin both for women with inherited thrombophilia and pregnancy complications and for those women with IVF-related implantation failure. Sufficiently powered randomised controlled trials are required to assess these outcomes before universal screening for thrombophilia in pregnancy can be justified.
